# An information-theoretic model for link prediction in complex networks

**DOI:** 10.1038/srep13707

**Published:** 2015-09-03

**Authors:** Boyao Zhu, Yongxiang Xia

**Affiliations:** 1 Department of Information Science and Electronic Engineering, Zhejiang University, Hangzhou 310027, China

## Abstract

Various structural features of networks have been applied to develop link prediction methods. However, because different features highlight different aspects of network structural properties, it is very difficult to benefit from all of the features that might be available. In this paper, we investigate the role of network topology in predicting missing links from the perspective of information theory. In this way, the contributions of different structural features to link prediction are measured in terms of their values of information. Then, an information-theoretic model is proposed that is applicable to multiple structural features. Furthermore, we design a novel link prediction index, called Neighbor Set Information (NSI), based on the information-theoretic model. According to our experimental results, the NSI index performs well in real-world networks, compared with other typical proximity indices.

The problem of link prediction aims at estimating the likelihood of the existence of a link in a given network on the basis of observed information^1^,^2^,^3^. Link prediction in complex networks has been studied by researchers in disparate scientific fields because of its significance in research and applications^4^,^5^,^6^,^7^,^8^,^9^,^10^. On the one hand, studies of link prediction have scientific significance. Link prediction could provide a useful method for evaluating the models that uncover the mechanisms that drive the growth and evolution of networks^4^. Although many network evolving models have been proposed to characterize the network evolving process^11^,^12^,^13^,^14^, it is very difficult to measure which model captures the real evolving process the most. Studies of link prediction inspired us to evaluate different evolving models by comparing the evolving likelihoods of the given network driven by these models^5^. In Ref. 6, the authors proposed the link predictability problem which characterizes the extent to which the links in a network can be predicted. Furthermore, an index called structural consistency was developed to numerically quantify the link predictability of networks. The study of link predictability can further help us evaluate link prediction algorithms and monitor sudden changes in the network evolving process. On the other hand, excellent link predictors have broad applications in different domains, such as checking possible protein-protein interactions in biological networks^7^,^8^, finding promising candidate friendships between users in social networks^9^, and providing personalized recommendations in E-commerce systems^10^.

The link prediction problem has received much attention in the field of network science^1^,^4^,^6^,^7^,^8^,^15^,^16^,^17^,^18^,^19^,^20^,^21^. Among various link prediction methods, the simplest framework is the set of similarity-based algorithms, where each node pair is assigned a score to estimate the similarity between two nodes^1^,^2^. These methods assume that the more similar two nodes are, the more likely the two nodes tend to be connected. Similarity-based methods can be further classified as node similarity-based methods and structure similarity-based methods^1^. The former supposes that two nodes sharing more common features tend to be connected^22^. However, entity’ attributes such as the user’s personal information in social networks may be unavailable for privacy reasons or unreliable for making predictions^1^. Compared with the attributes of nodes, the structural features of networks are easier to obtain and more reliable. Hence, similarity-based algorithms in complex networks mainly focus on structural similarity. A wealth of algorithms based on structural similarity have been proposed in the past years. For example, Common Neighbors (CN) is a basic index based on local network structural properties but has a relatively high prediction accuracy^15^. Indices that are the variants of the CN index, such as Adamic-Adar (AA)^23^, Resource Allocation (RA)^15^, are called CN-based methods. Many other structural similarity-based methods have also been designed to estimate the similarity of nodes^24^,^25^,^26^,^27^. Moreover, many algorithms based on maximum-likelihood methods^7^,^8^,^17^ and probabilistic models^28^ have also been proposed. For the hierarchical structure of networks, Clauset *et al.* proposed a Hierarchical Structure Model that estimates the connection likelihood by using a dendrogram^8^. Guimerà *et al.* developed a Stochastic Block Model to capture the community structure and estimate the probability that two nodes are connected^7^. Liu *et al.* recently proposed a Fast Blocking probabilistic Model based on a greedy strategy, which can reduce the computation complexity and improve the prediction accuracy^17^. In this model, link likelihoods are estimated by considering link densities within and among communities. Friedman *et al.* developed a Probabilistic Relational Model to handle the cases in which databases are relational^28^.

Thus far, the aim of previous proposed frameworks has been to quantify the likelihood of candidate links existing. In other words, the problem of link prediction can be treated as predicting the likelihood of the event that two nodes are connected. In information theory^29^,^30^, the information quantifies the uncertainty associated with the outcome of a random variable or an event. Hence, the link likelihood between a pair of nodes can be estimated by the information from the viewpoint of information theory. Recently, Tan *et al.* proposed a Mutual Information method which can significantly enhance the prediction accuracy in large networks^31^. In the Mutual Information index, the feature of common neighbors is considered to facilitate prediction and the link likelihood of a node pair is denoted as the conditional self-information of the event that the node pair is connected when their common neighbors are given.

In fact, any structural feature of a network can provide information to facilitate link prediction. Based on this idea, we develop an information-theoretic model for link prediction, which is applicable to various structural features. The Mutual Information approach^31^ can be considered as an example of this model when only one feature, i.e., common neighbors, is considered. Furthermore, the proposed model can also handle the cases in which multiple structural features are available. As an example, we design a novel link prediction index called Neighbor Set Information (NSI), which uses two types of local structural features. We test the NSI index in twelve real-world networks and find that it performs well compared with other structure based indices.

## Results

### An information-theoretic model for link prediction

In previous studies, different structural features have been used to facilitate link prediction. Two typical examples of structural features are common neighbors of a node pair and community structure in a network. However, most previous prediction algorithms focus only on one or two structural features. If many features are given at the same time, there is no good way to benefit from all of the information available. In our information-theoretic model, in contrast, any structural feature can be used to provide information to facilitate link prediction, and the information from different features can be combined easily. In this sense, the proposed method can make better use of all of the information available.

We begin with the case where just one feature is available. For a feature *F* associated with the candidate node pair, the set of feature variables is denoted as Ω, and *ω* is one feature variable of Ω. For example, if we choose the common neighbors of a node pair (*x, y*) as the available feature *F*, then the variable set Ω is denoted as 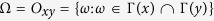, where Γ(*x*) is the neighbor set of node *x* and ω is one common neighbor of node pair (*x, y*).

Given a disconnected node pair (*x, y*) and one feature *F* associated with (*x, y*), the event of node pair (*x, y*) being connected is denoted as 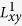. Hence, the link prediction problem can be described as estimating the uncertainty of event 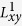 from the information supplied by feature *F*. According to information theory^29^,^30^ (please refer to the Supplementary Information (SI) for details), the existence likelihood of a link can be estimated by the *conditional self-information* which is defined as
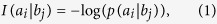


where *a*_*i*_ and *b*_*j*_ are two events that belong to event sets *A* and *B*, respectively, and *p*(*a*_*i*_|*b*_*j*_) is the probability that event *a*_*i*_ happens given that event *b*_*j*_ has already happened. The conditional self-information indicates the uncertainty of event *a*_*i*_ when event *b*_*j*_ is given.

According to the above definition, for the link prediction task, the likelihood score can be defined as
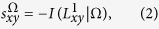
where 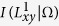 indicates the conditional self-information of the connection of node pair (*x, y*) when feature variable set Ω is available. According to its definition^29^, the smaller 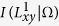 is, the higher the probability of a link between nodes *x* and *y* tends to be. Therefore, we define the score as the negation of 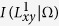. If the feature variables in Ω are assumed to be independent of each other, then
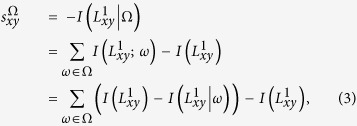
where 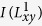 is the self-information of the event that node pair (*x, y*) is connected, and 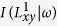 denotes the conditional self-information of the event that node pair (*x, y*) is connected when a feature variable *ω* is known (please refer to the SI for a detailed derivation). Because we are primarily focusing on the structural properties of the network, 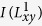 and 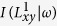 can be calculated by the statistical structural properties. It should be noted that feature *F* is not specified in the algorithm and can be any structural feature that we can obtain from the network.

What we have considered above is the case in which only one feature of the network is obtained. In practice, various features may be available, and they may all be helpful for link prediction. However, different features show different aspects of network structural properties. For example, shortest path and clustering are features that are commonly used in link prediction. Most nodes in networks are connected by a very short distance^2^, which characterizes the famous “small world” property of networks. On the other hand, clustering indicates that a node with a dense neighborhood is more likely to have more links than one with a sparse neighborhood. Although both of these features are helpful to predict missing links, the properties they reflect are different. In this case, there is no direct way for traditional link prediction algorithms to simultaneously make good use of both features at the same time. In contrast to those algorithms, we use the value of information to evaluate the connection likelihood. The effects of structural features on prediction are unified to the values of conditional self-information. Hence, even with different features, the values of information brought by these features are additive. Therefore, Eq. (3) can be easily extended to the case of multiple features. Under this condition, the variable set for feature *i* is denoted as Ω_*i*_. Then, we adopt a parameter λ_*i*_ to reckon the contribution of feature *i* to the final connection likelihood, and define the likelihood score as
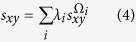


Altogether, we obtain the information-theoretic model to evaluate the connection likelihood when any structural feature is given. In this sense, Ref. 31 can be considered as an example of our model for which only the feature of common neighbors is applied.

### An information-theoretic approach based on neighbor set

In this subsection, we will introduce an information-theoretic approach based on neighbor set, as an example of the application of our information-theoretic model.

**Figure 1:**
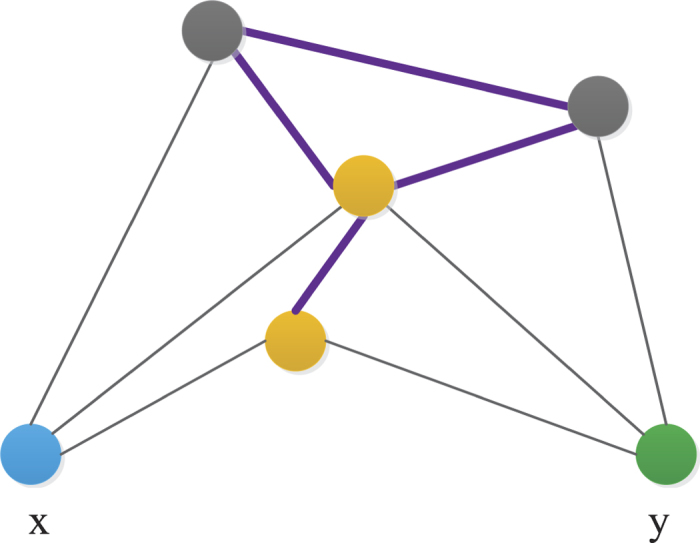
Illustration of the relationship between two neighbor sets. The neighbors of node *x* and node y can be denoted as neighbor set *x* and neighbor set *y*, respectively. There are two common neighbors between neighbor sets *x* and *y*, and these are colored in yellow. Lines emphasized in purple describe the links across two neighbor sets. The “connections” between two neighbor sets are mainly divided into two categories: common neighbors and links across two neighbor sets.

The neighbor set of node *x* is defined as the node set consisting of the neighbors of node *x*, i.e., Γ(*x*). For a candidate node pair (*x, y*), our fundamental hypothesis is that the more strongly their neighbor sets are connected, the more likely they are connected. The link likelihood of two nodes can thus be estimated by the information brought by the “connections” between their neighbor sets. In particular, the “connections” involve two categories: overlap nodes of two neighbor sets, i.e., common neighbors of the candidate node pair, and the links across two neighbor sets. Formally, the common neighbors of node pair (*x, y*) are denoted as 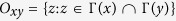 and the links across neighbor sets Γ(*x*) and Γ(*y*) are defined as 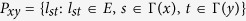, where *E* denotes the link set of the network. Both features are helpful for predicting missing links. In social networks, for instance, the neighbor set of node *x* denotes the friends associated with *x*. If two people have many common friends, or if their friends are also mutual friends, these two people are more likely to be friends in the future. This agrees well with our intuition. In Fig. 1, an example is provided to further illustrate the relationship of two neighbor sets.

Based on the motivation described above, the information given by the features extracted from the “connections” between two neighbor sets, i.e., the common neighbors and the links across two neighbor sets, is used to facilitate link prediction. According to the information-theoretic model described in Eq. (4), the link likelihood of a node pair is defined as
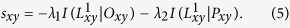
From this equation, the score can be locally calculated by the neighbor sets of nodes *x* and *y* based on the information-theoretic model, so we call it the Neighbor Set Information (NSI) index (please refer to the SI for the detailed derivation). For a simpler formalization, we define the ratio *λ* as *λ*_2_/*λ*_1_, and obtain
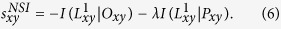


To demonstrate the performance of the NSI index, twelve networks from disparate fields are considered in our experiments (see SI for details). Two widely used metrics called *area under the receiver operating characteristic curve* (AUC)^32^ and *Precision*^33^ are considered to evaluate the accuracy of the link prediction algorithms (please refer to the Methods section for details). Indices for comparison are summarized in the Methods section. The prediction accuracy results are presented in Tables 1 and 2.

**Figure 2:**
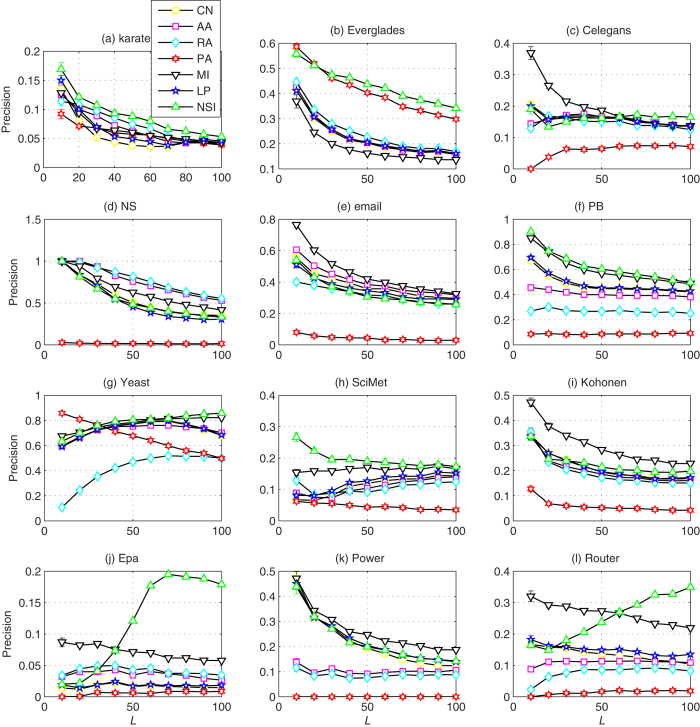
Illustration of the dependence of Precision on the number of L. The horizontal axis denotes that *top-L* links are used for the evaluation of Precision. Each value of Precision is a result averaged over 100 independent implementations, and the error bars represent the standard deviations. The parameters of the NSI and LP indices are typically fixed as *λ* = 0.1 and *ε* = 0.001, respectively.

Table 1 shows the prediction accuracy measured by AUC. According to the AUC results, our NSI index performs the best or nearly the best in most networks. Because the AA index and RA index are variants of CN, they have nearly the same AUC values in most networks. The PA and MI indices provide better prediction accuracy in EPA and Router, while in other networks, they perform worse than the NSI index. Compared with LP, the NSI index always performs better (or at least the same). In contrast with AUC, the Precision metric focuses on the most likely latent links. According to Table 2, the NSI index achieves competitive performance in most networks. In the definition of Precision, its value depends on the number of *top-L* candidate links to be predicted. Here, we also investigate the dependence of Precision on the number of *L* and present the results in Fig. 2. For the convenience of comparison, the parameter *ε* of LP is set as 0.001^34^ and the ratio *λ* of NSI is fixed as 0.1. From the results in Fig. 2, we find that although *L* changes, the NSI index can achieve a high Precision accuracy in most networks, especially in SciMet and Epa. Combining the results above, the NSI index has the overall best performance regardless of whether the metric used is AUC or Precision.

**Figure 3:**
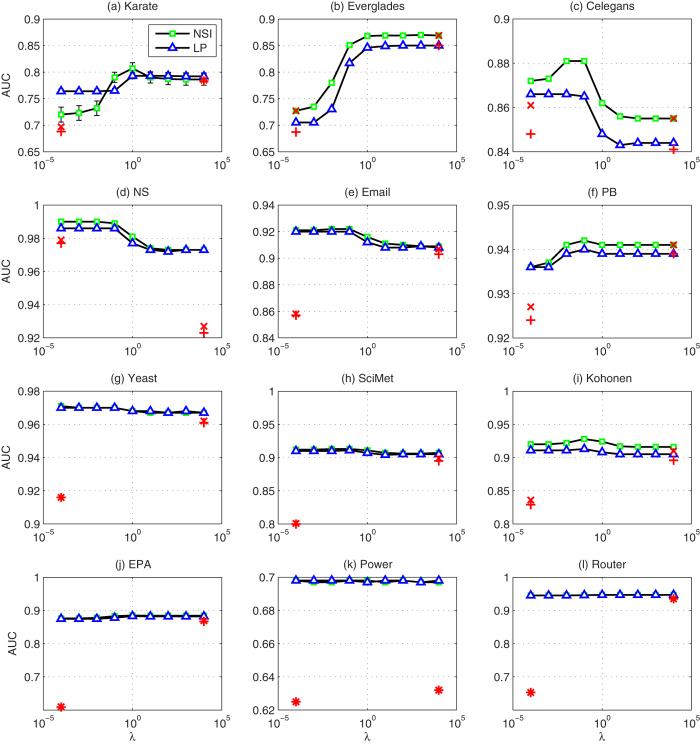
AUC results of the LP and NSI indices in twelve networks. Each AUC value is averaged over 100 independent implementations, and the error bars represent the standard deviations (please refer to Table 1 for the detailed values). The parameters of the LP and NSI indices range from 10^−4^ to 10^4^ with a ratio of 10. The red cross marks represent the performance of the features of common neighbors (left cross mark) and links across two neighbor sets (right cross mark) separately applied by the information-theoretic model. The red plus marks stand for the performance of the indices of paths with length 2 (left plus mark) and 3 (right plus mark).

**Figure 4:**
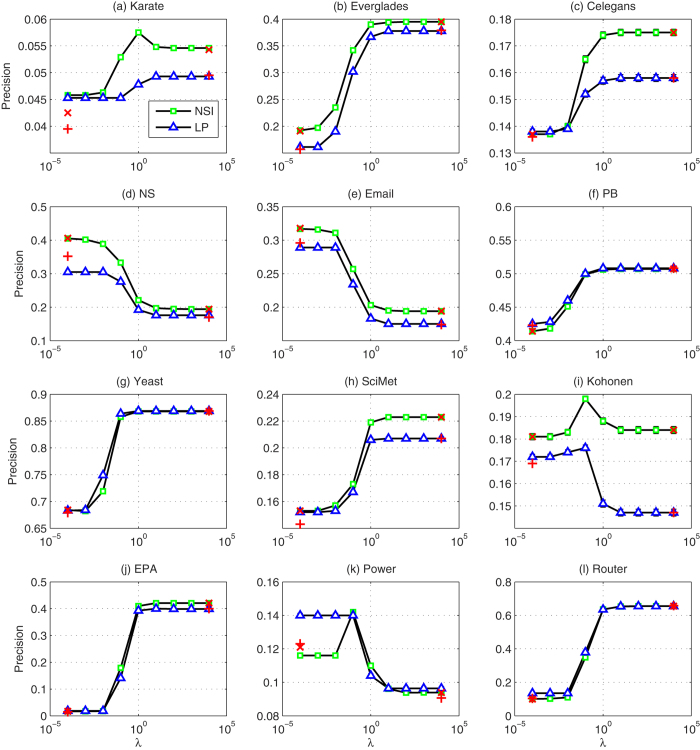
Precision results of the LP and NSI indices in twelve networks. Each Precision value is averaged over 100 independent implementations, and the error bars represent the standard deviations (please refer to Table 2 for the detailed values). The parameters of the LP and NSI indices range from 10^−4^ to 10^4^ with a ratio of 10. The red cross marks represent the performance of the features of common neighbors (left red cross mark) and links across two neighbor sets (right cross mark) separately applied by the information-theoretic model. The red plus marks stand for the performance of the indices of paths with length 2 (left plus mark) and 3 (right plus mark).

Because the performance of NSI depends on the ratio *λ*, we plot the AUC and the Precision accuracy of NSI index as functions of the ratio *λ*. In Figs. 3 and 4, although the prediction performance of NSI index changes with different trends in different networks when *λ* changes, we find that *λ* = 0.1 always produces a reasonable performance in the twelve real-world networks considered. In Tables 1 and 2, we list the performance of the NSI index with a fixed ratio *λ* = 0.1, and find that it performs well compared with six other typical proximity indices. Therefore, the NSI index is highly valuable for applications because one can directly set *λ* to a fixed value, rather than searching for its optimal value, which in practice takes a significant amount of time.

## Discussion

In this paper, we develop an information-theoretic model that treats the link prediction problem as the evaluation of the uncertainty that a link exists. Furthermore, the proposed model is applicable to various structural features and can address the case in which multiple features are available.

The information-theoretic model has two advantages. The first is that, in contrast to traditional link prediction methods, the information-theoretic model evaluates the link likelihood via the value of information. Even for features that belong to different structural properties, the values of information brought by these features are additive. In this way, the proposed model can easily make use of diverse features that are available. Although some indices, such as the LP index, can use more than one feature to make predictions, the chosen features often belong to the same type of structural property. Thus, the information-theoretic model can take advantage of all of the available features to make a better prediction. The second advantage is that, when focusing on one feature of the network, the values of information provided by different feature variables are still distinguishable. To obtain a better understanding, we return to Eq. (3), as it is used to calculate the contribution of each feature to the connection likelihood. In this equation, 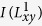 is the prior information, which has nothing to do with the feature. Hence, the effect of the chosen feature on the connection likelihood is given by 
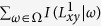
. Although the feature variables are extracted from the same feature of the network, their contributions to the value of information can be different, i.e., 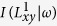 can be different for different *ω*. Actually, we can find similar settings in many other good link prediction methods. For example, the AA and RA indices differentiate the effects of different common neighbors by considering their degrees. This is a wise method to make the prediction more accurate. In summary, the information-theoretic model can make use of different features, and with each feature, it can differentiate the contributions of different variables. Therefore, it can achieve good prediction accuracy.

To illustrate the above advantages more clearly, we take the NSI index as an example. For the NSI index, the features of common neighbors and links across two neighbor sets are used. First, we show how the use of two features facilitates link prediction. The performance of the NSI index is given in Figs. 3 (for AUC) and 4 (for Precision) as functions of the free parameter *λ*. For comparison, in these figures, we also plot the results when only one of the two features is considered. The figures display the special cases when *λ* = 0 and *λ* → ∞, respectively. We find that the use of two features provides better results or at least similar results, than the use of only one feature in most cases.

**Figure 5:**
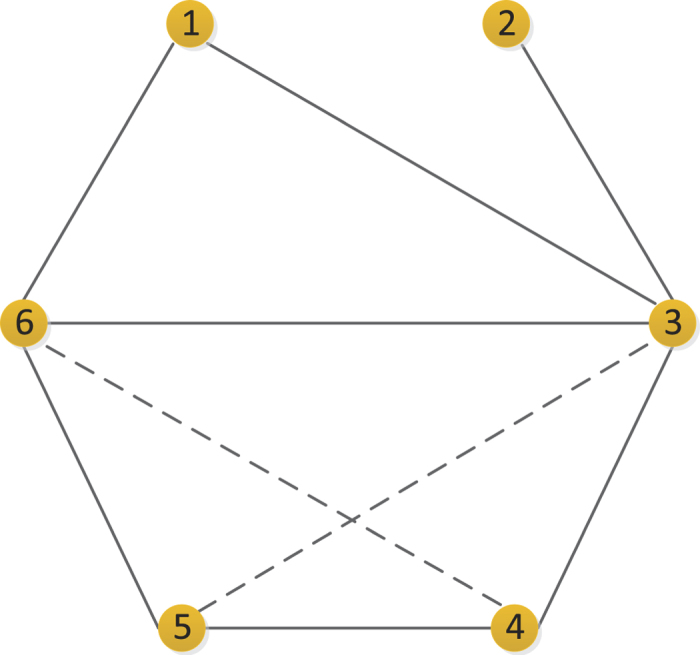
Illustration of an example network. Two dashed lines denote two possible links of node pair (3, 5) and node pair (4, 6). Measured by the LP index, they all have the same score because node pair (3, 5) and node pair (4, 6) both have two common neighbors and one path with length 3. For the NSI index, node 3 and node 6 have different contributions in that the clustering coefficient of node 3 is 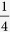 and the clustering coefficient of node 6 is 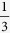, which means that the contribution of node 6 to the connection of node pair (3, 5) is greater than that of node 3 to node pair (4, 6).

In addition, for comparison, we plot the performance of the LP index in Figs. 3 and 4. Because two features are used in LP, a similar free parameter *ε* is considered to measure the contributions of the two features. We find that the NSI index performs better than LP in almost all of the cases considered. This result demonstrates the second advantage of our information-theoretic model, i.e., this model differentiates the impact of the feature variables on the connection likelihood via 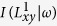. More specifically, in the NSI index, we distinguish the contribution of each node and link through 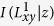 and 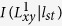, respectively, whereas in the LP index, they are treated equally. In Fig. 5, we provide an example to further describe this effect. Node pairs (3, 5) and (4, 6) which are marked by dashed lines, are two links to be predicted. According to the definition of the LP index, these two possible links are indistinguishable because Eq. (18) produces the same score for both of them. However, by using the NSI index, the score for node pair (3, 5) is higher than that for node pair (4, 6), which means that node pair (3, 5) is more likely to be linked. This result is a good fit for the clustering mechanism^35^ in network evolving process. Clearly, the setting of NSI is more reasonable and is closer to the real case. As a result, we find that the performance of the NSI index is better than that of the LP index.

## Methods

### Link prediction algorithm

Consider an undirected network *G*(*V, E*), where *V* and *E* are the node set and link set, respectively. Self-links and multi-links are not allowed. For each non-existent link, e.g., *l*_*xy*_ ∈ *U−E*, where *x, y* ∈ *V* and *U* denotes the universal possible link set, our task is to assign a score to estimate its connection likelihood. Note that we do not differentiate connection likelihood and score here. If a rank for all non-observed links is given, for the most likely candidate links, one can choose the links with the highest ranks.

Given a predictor we can rank all of the non-observed links according to the scores they obtained. To validate the prediction performance of a predictor, the observed links of the network are randomly divided into two parts, i.e., the training set *E*^*T*^ and the probe set *E*^*P*^. Here, *E*^*T*^ is treated as known information while *E*^*P*^ is only used to test algorithms. Clearly, we have *E*^*T*^ » *E*^*P*^ = *E* and *E*^*T*^ « *E*^*P*^ = Ø. In this paper, the fraction of links in the training set is 90% and the remainder constitutes the probe set.

### Evaluation metrics

In this study, we apply two widely used metrics called *area under the receiver operating characteristic curve* (AUC)^32^ and *Precision*^33^ to evaluate the accuracy of the link prediction algorithms.

• AUC can be interpreted as the probability that a randomly chosen missing link (link in *E*^*P*^) has a higher score than a randomly chosen non-existent link (link in *U*−*E*). In real implementations, among *n* times of interdependent comparisons, if there are *n*′ times in which the score of the missing link is higher than that of the non-existent link and *n*″ times in which the two have the same score, the AUC value can be expressed as

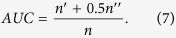

- If all of the scores are generated from an independent and identical distribution, the AUC value should be approximately 0.5. Therefore, the extent to which AUC exceeds 0.5 indicates how much better the algorithm performs than pure chance.
• Precision focuses on top-ranked latent links, while AUC considers the macroscopic accuracy. Each non-observed link is given a score, and we sort these scores in descending order. If there are *L*_*r*_ relevant links in *E*^*P*^, when we choose the *top-L* links, then

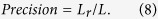

- Clearly, higher Precision means higher accuracy.

### Benchmarks

Here, six typical proximity indices are considered for performance comparisons, including Common Neighbors (CN)^15^, Adamic-Adar (AA)^23^, Resource Allocation (RA)^15^, Preferential Attachment (PA)^35^ Mutual Information (MI)^31^ and Local Path (LP)^15^.

The CN index assumes that two nodes sharing more common neighbors tend to be connected. It is defined as
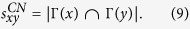


The AA index supposes that the larger degree of the common neighbor, the less weight it can contribute. Formally, it is denoted as
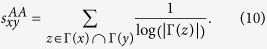


The RA index is similar to AA, but is motivated by the process of resource allocation. The penalty for a high degree common neighbor is more sufficient in RA than it is in AA. The score is defined as
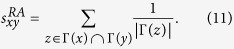


Originating from the network evolving mechanism, the PA index supposes that the probability that two nodes are connected is proportional to the product of their degrees. Thus, it is defined as
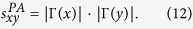


The MI index estimates the effect of common neighbors on the link probability via information theory. In the MI index, the prior probability that node pair (*x, y*) is connected can be calculated by
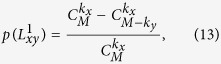
where *k*_*x*_ and *k*_*y*_ are the degrees of nodes *x* and *y*, respectively, and *M* is the number of links in the training set. Thus, the likelihood score can be described as
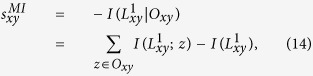
where 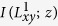 is estimated by
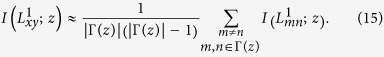

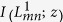
 can be further derived as
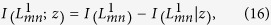
where 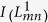 can be calculated from Eq. (13). In particular, 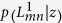 can be estimated by the clustering coefficient of node *z*, which is denoted as
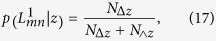


where *N*_Δ*z*_ and 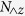 are respectively the numbers of connected and disconnected node pairs whose common neighbors include node *z*.

The LP index considers the information regarding the next nearest neighbors, which can remarkably enhance the prediction accuracy. It is described as
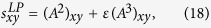
where *ε* is a free parameter. (*A*^2^)_*xy*_ and (*A*^3^)_*xy*_ are the numbers of different paths with length 2 and 3 connecting *x* and *y*, respectively.

## Additional Information

**How to cite this article**: Zhu, B. and Xia, Y. An information-theoretic model for link prediction in complex networks. *Sci. Rep.* **5**, 13707; doi: 10.1038/srep13707 (2015).

## Supplementary Material

Supplementary Information

## Figures and Tables

**Table 1:** Comparison of the prediction accuracy under the AUC metric in twelve networks.

Networks	*CN*	*AA*	*RA*	*PA*	*MI*	*NSI*_*0.1*_	LP^*^	NSI^*^
Karate	0.697(52)	0.733(68)	0.74(71)	0.727(68)	0.736(68)	0.79(98)	0.793(57)	**0.807(106)**
Everglades	0.687(5)	0.695(5)	0.703(5)	0.813(5)	0.512(5)	0.851(5)	0.85(5)	**0.87(5)**
Celegans	0.848(2)	0.865(2)	0.869(1)	0.755(3)	0.84(2)	**0.881(1)**	0.866(1)	**0.881(1)**
NS	0.977(1)	0.98(1)	0.981(1)	0.659(8)	0.927(5)	0.989(0)	0.986(0)	**0.99(0)**
Email	0.857(1)	0.859(1)	0.858(1)	0.805(1)	0.892(1)	**0.922(1)**	0.92(1)	**0.922(1)**
PB	0.924(0)	0.927(0)	0.929(0)	0.909(0)	0.932(0)	**0.942(0)**	0.94(0)	**0.942(0)**
Yeast	0.916(0)	0.917(0)	0.917(0)	0.864(0)	0.936(0)	0.97(0)	0.97(0)	**0.971(0)**
SciMet	0.8(0)	0.802(1)	0.801(0)	0.807(1)	0.871(1)	**0.913(0)**	0.911(0)	**0.913(0)**
Kohonen	0.829(0)	0.836(0)	0.836(0)	0.866(0)	0.912(0)	**0.928(0)**	0.913(0)	**0.928(0)**
EPA	0.609(0)	0.61(0)	0.611(0)	0.915(0)	**0.926(0)**	0.883(1)	0.883(1)	0.885(0)
Power	0.625(1)	0.625(1)	0.625(1)	0.578(1)	0.609(1)	**0.698(1)**	**0.698(1)**	**0.698(1)**
Router	0.653(1)	0.654(1)	0.653(1)	0.956(0)	**0.957(0)**	0.946(0)	0.947(0)	0.947(0)

Each value is obtained by averaging over 100 implementations with independent random divisions of the training set (90%) and the probe set (10%). The numbers in the brackets denote the standard deviations. For example, 0.697(52) means that the AUC value is 0.697 and the standard deviation is 52 × 10^−4^. *NSI*_0.1_ denotes the NSI index with a fixed ratio of *λ* = 0.1. The best performance is emphasized by bold font. The last two columns show the best performance of the LP and NSI indices shown in Fig. 3.

**Table 2:** Comparison of the prediction accuracy under the Precision (Top-100) metric in twelve networks.

Networks	*CN*	*AA*	*RA*	*PA*	*MI*	*NSI*_0.1_	LP^*^	NSI^*^
Karate	0.0395(2)	0.0426(1)	0.0427(1)	0.0385(2)	0.043(2)	0.0529(2)	0.0493(2)	**0.0575(2)**
Everglades	0.157(6)	0.158(6)	0.169(8)	0.298(13)	0.136(6)	0.342(14)	0.378(12)	**0.395(11)**
Celegans	0.136(9)	0.135(10)	0.125(10)	0.0711(7)	0.137(10)	0.165(13)	0.158(13)	**0.175(12)**
NS	0.352(23)	0.53(18)	**0.553(15)**	0.0139(1)	0.416(24)	0.333(23)	0.305(36)	0.405(22)
Email	0.296(19)	0.319(17)	0.261(15)	0.0297(3)	**0.325(16)**	0.257(18)	0.289(17)	0.317(18)
PB	0.423(14)	0.381(13)	0.251(11)	0.0921(8)	0.488(15)	0.499(20)	**0.508(20)**	0.507(20)
Yeast	0.679(39)	0.701(29)	0.499(18)	0.497(32)	0.822(5)	0.858(2)	**0.869(0)**	0.868(0)
SciMet	0.143(10)	0.14(10)	0.124(10)	0.035(3)	0.166(10)	0.173(13)	0.207(13)	**0.223(16)**
Kohonen	0.169(12)	0.161(12)	0.151(13)	0.0423(3)	**0.228(17)**	0.198(13)	0.176(13)	0.198(13)
EPA	0.0147(1)	0.0266(2)	0.0345(2)	0.0086(1)	0.0577(4)	0.179(9)	0.4(28)	**0.421(37)**
Power	0.123(10)	0.106(7)	0.0873(8)	0(0)	**0.187(12)**	0.142(12)	0.14(13)	0.142(12)
Router	0.104(7)	0.109(6)	0.0822(6)	0.0185(1)	0.219(13)	0.349(12)	**0.655(27)**	**0.655(33)**

Each value is obtained by averaging over 100 implementations with independent random divisions of the training set (90%) and the probe set (10%). The numbers in the brackets denote the standard deviations. For example, 0.0395(2) means that the Precision value is 0.0395 and the standard deviation is 2 × 10^−4^. *NSI*_0.1_ denotes the NSI index with a fixed ratio of λ = 0.1. The best performance is emphasized by bold font. The last two columns show the best performance of the LP and NSI indices shown in Fig. 4.
