# Supplementary material for: An information-theoretic model for link prediction in complex networks
Source: Sci Rep. 2015 Sep 3;5:13707. doi: 10.1038/srep13707 (PMC4558573; doi:10.1038/srep13707)
Supplement: Supplementary Information [file srep13707-s1.pdf]

An information-theoretic model for link prediction in complex networks

Boyao Zhu & Yongxiang Xia

Supplementary Information

A Brief Introduction to Information Theory

Information theory was developed by Claude E. Shannon [1] to find fundamental limits on signal processing operations, which has a broad application in electrical engineering, applied mathematics and computer science. In information theory, information is associated with the uncertainty of an event or a random variable. For example, the event that it will rain tomorrow is not sure for us. Thus, the information is defined as the value to reduce the uncertainty of an event or a valuable. Formally, we have the following definitions.

The uncertainty of an outcome can be measured by the self-information.

Definition 1 For a random variable X, an outcome x_k occurs with the probability $p(x_k)$, then the *self-information* $I(x_k)$ can be denoted as [1]

$$I(x_k) = \log \frac{1}{p(x_k)} = -\log p(x_k), \tag{1}$$

where the base of the logarithm is specified as 2.

The self-information indicates the uncertainty of the outcome x_k. It is related to the probability $p(x_k)$. Clearly, the higher the self-information is, the lower probability the outcome occurs. Similarly, if we have the conditional probability $p(x_i|y_j)$, we can define the *conditional self-information* as

$$I(x_i|y_j) = -\log p(x_i|y_j). \tag{2}$$

$I(x_i|y_j)$ indicates the uncertainty of the outcome x_i when the outcome y_j is given. When x_i and y_j are independent to each other, $I(x_i|y_j)$ equals to $I(x_i)$.

Definition 2 Considering two variables X and Y with a joint probability distribution function $p(x, y)$ and marginal probability distribution functions $p(x)$ and $p(y)$. The *mutual information* $I(X;Y)$ is defined as [2]

$$\begin{aligned} I(X;Y) &= \sum_{x\in X}\sum_{y\in Y} p(x,y)\log\frac{p(x,y)}{p(x)p(y)} \\ &= \sum_{x,y} p(x,y)\log\frac{p(x|y)}{p(x)}. \end{aligned} \tag{3}$$

Therefore, the mutual information $I(x_i; y_j) = I(X = x_i; Y = y_j)$ can be denoted as

$$\begin{aligned} I(x_i;y_j) &= \log\frac{p(x_i|y_j)}{p(x_i)} \\ &= -\log p(x_i) - (-\log p(x_i|y_j)) \\ &= I(x_i) - I(x_i|y_j). \end{aligned} \tag{4}$$

The mutual information gives the reduction in uncertainty of an outcome x_i when the outcome of another variable y_j is given.

As to the link prediction problem, we primarily estimate the probability that the node pairs are connected based on some prior known information. For a node pair (x, y), if the event that node pair (x, y) is connected is denoted as L^1_{xy}, the self-information of event L^1_{xy} is $I(L^1_{xy}) = -\log p(L^1_{xy})$, where $p(L^1_{xy})$ is the prior probability of the connection of node pair (x, y). If the common neighbors of node pair (x, y) are known, then the uncertainty of the event L^1_{xy} reduces. Formally, the common neighbors of node pair (x, y) is defined as $O_{xy} = \{z : z \in \Gamma(x) \cap \Gamma(y)\}$, where $\Gamma(x)$ is the neighbors of node x. Then the conditional self-information of event L^1_{xy} when the common neighbors are given is $I(L^1_{xy}|O_{xy})$. The higher value of $I(L^1_{xy}|O_{xy})$ means the event L^1_{xy} is less likely to happen. Based on this idea, we could distinguish which link is more likely to be formed.

The Derivation of the Information-theoretic Model

For the case with only one topological feature. Given a disconnected node pair (x, y) and one feature F associated with (x, y). When obtaining the feature variable set Ω corresponding to feature F, the probability score can be defined as

$$s^{\Omega}_{xy} = -I(L^1_{xy}|\Omega), \tag{5}$$

According to the definition of mutual information [2], $I(L^1_{xy}|\Omega)$ can be denoted as

$$I(L^1_{xy}|\Omega) = I(L^1_{xy}) - I(L^1_{xy}; \Omega), \tag{6}$$

where $I(L^1_{xy})$ is the value of self-information of that node pair (x, y) is connected, and $I(L^1_{xy}; \Omega)$ denotes the value of mutual information between the event that node pair (x, y) is connected and the event that feature variable set Ω is available, which indicates the reduction in uncertainty of the connection between nodes x and y when feature variable set Ω is given.

If the feature variables in Ω are assumed to be independent to each other, then

$$I(L^1_{xy}; \Omega) = \sum_{\omega \in \Omega} I(L^1_{xy}; \omega). \tag{7}$$

In the real implementation, this assumption depends on the chosen feature, and it is true in most cases. For example, common neighbors in the set $\Omega = O_{xy} = \{\omega : \omega \in \Gamma(x) \cap \Gamma(y)\}$ are independent to each other.

$I(L^1_{xy}; \omega)$ can be further derived as

$$I(L^1_{xy}; \omega) = I(L^1_{xy}) - I(L^1_{xy}|\omega), \tag{8}$$

where $I(L^1_{xy}|\omega)$ is the conditional self-information of that node pair (x, y) is connected when a feature variable ω is known.

We substitute Eqs. (6), (7) and (8) into Eq. (5) and obtain

$$\begin{aligned} s^{\Omega}_{xy} &= -I(L^1_{xy}|\Omega) \\ &= \sum_{\omega \in \Omega} I(L^1_{xy}; \omega) - I(L^1_{xy}) \\ &= \sum_{\omega \in \Omega} (I(L^1_{xy}) - I(L^1_{xy}|\omega)) - I(L^1_{xy}). \end{aligned} \tag{9}$$

Consider the case with multiple topological features, the variable set for feature i is denoted as Ω_i. Then, we adopt a parameter λ_i to reckon the contribution of feature i to the final connection likelihood, and define the probability score as

$$s_{xy} = \sum_i \lambda_i s^{\Omega_i}_{xy}, \tag{10}$$

The Neighbor Set Information Approach to Link Prediction

In this section, we will introduce an information-theoretic approach based on the neighbor set in details.

As shown in Fig. 1, the features we considered here are the feature of common neighbors and the feature of link across two neighbor sets. For a disconnected node pair (x, y), the set of common neighbors is denoted as $O_{xy} = \{z : z \in \Gamma(x) \cap \Gamma(y)\}$ and the set of links across two neighbor sets is defined as $P_{xy} = \{l_{st} : l_{st} \in E, s \in \Gamma(x), t \in \Gamma(y)\}$, where E denotes the link set of the network and $\Gamma(x)$ is the neighbor set of node x.

First, let's discuss the information caused by the common neighbors. If the event of the connection of node pair (x, y) is described as L^1_{xy}, denoted by $p(L^1_{xy})$, the prior probability of the connection of node pair (x, y) can be defined as

$$p(L^1_{xy}) = \frac{|E^T|}{|V|(|V|-1)/2}, \tag{11}$$

which indicates the link density in the training set. According to the information theory [1,2], the effect of common neighbors on the connection of two nodes can be estimated by

$$I(L^1_{xy}|O_{xy}) = I(L^1_{xy}) - I(L^1_{xy}; O_{xy}), \tag{12}$$

where $I(L^1_{xy})$ is the self-information of that node pair (x, y) has one link. $I(L^1_{xy}; O_{xy})$ is the mutual information between the event that node pair (x, y) is connected and the event that the common neighbor set O_{xy} is known. $I(L^1_{xy}; O_{xy})$ indicates the reduction in uncertainty of the connection between node x and node y when the common neighbors are available.

If the nodes of O_{xy} are assumed to be independent of each other, then

$$I(L^1_{xy}; O_{xy}) = \sum_{z \in O_{xy}} I(L^1_{xy}; z), \tag{13}$$

where z is one of the common neighbors of node x and node y. According to the definition of mutual information, $I(L^1_{xy}; z)$ can be wrote as

$$I(L^1_{xy}; z) = I(L^1_{xy}) - I(L^1_{xy}|z). \tag{14}$$

Particularly, $p(L^1_{xy}|z)$ is the clustering coefficient of node z, and can be denoted as

$$p(L^1_{xy}|z) = \frac{N_{\triangle z}}{N_{\triangle z} + N_{\wedge z}}, \tag{15}$$

where $N_{\triangle z}$ and $N_{\wedge z}$ are respectively the numbers of connected and disconnected node pairs whose common neighbors include node z.

We substitute Eq. (13) into Eq. (12) and obtain

$$I(L^1_{xy}|O_{xy}) = I(L^1_{xy}) - \sum_{z \in O_{xy}} I(L^1_{xy}; z), \tag{16}$$

where $I(L^1_{xy})$ and $I(L^1_{xy}; z)$ can be calculated by Eqs (11) and (14), respectively.

Moreover, the information brought by the links across two neighbor sets can also be used to make predictions. The effect of links between two neighbor communities on the connection likelihood can be estimated by

$$I(L^1_{xy}|P_{xy}) = I(L^1_{xy}) - I(L^1_{xy}; P_{xy}), \tag{17}$$

where $I(L^1_{xy}; P_{xy})$ is the mutual information between the event that node pair (x, y) is connected and the event that the links cross neighbor sets of nodes x and y are known. $I(L^1_{xy}; P_{xy})$ denotes the reduction

of uncertainty in the connection of node pair (x, y) when the links between neighbor sets of nodes x and y are obtained.

If the links in P_{xy} are supposed to be independent of each other, then

$$I(L^1_{xy}; P_{xy}) = \sum_{l_{st} \in P_{xy}} I(L^1_{xy}; l_{st}), \tag{18}$$

where l_{st} is a link in P_{xy} with endpoints s and t. In addition, $I(L^1_{xy}; l_{st})$ can be denoted as

$$I(L^1_{xy}; l_{st}) = I(L^1_{xy}) - I(L^1_{xy}|l_{st}), \tag{19}$$

where $I(L^1_{xy}|l_{st})$ is the conditional self-information of the event that node pair (x, y) is connected when link (s, t) is the link between neighbor sets of nodes x and y. Here $p(L^1_{xy}|l_{st})$ can be calculated in a similar way of estimating $p(L^1_{xy}|z)$, which can be described as

$$p(L^1_{xy}|l_{st}) = \frac{N_{\square st}}{N_{\square st} + N_{\sqcap st}}, \tag{20}$$

where $N_{\square st}$ stands for the number of connected node pairs whose neighbors are s and t respectively and $N_{\sqcap st}$ denotes the number of disconnected node pairs whose neighbors are s and t respectively.

We substitute Eq. (18) into Eq. (17), and obtain

$$I(L^1_{xy}|P_{xy}) = I(L^1_{xy}) - \sum_{l_{st} \in P_{xy}} I(L^1_{xy}; l_{st}), \tag{21}$$

where $I(L^1_{xy})$ and $I(L^1_{xy}; l_{st})$ can be calculated by Eqs. (11) and (19) respectively.

Now given a disconnected node pair (x, y), we can obtain the common neighbors and links across the node pair's neighbor sets. After calculating the effect of common neighbors and links between the node pair's neighbor sets using Eqs. (16) and (21) respectively, the connection likelihood score for this non-adjacent node pair can be defined as

$$s_{xy} = -\lambda_1 I(L^1_{xy}|O_{xy}) - \lambda_2 I(L^1_{xy}|P_{xy}). \tag{22}$$

According to this equation, the score can be locally calculated by the neighbor sets of nodes x and y by using the information-theoretic model. Thus, we call it Neighbor Set Information (NSI) index. According to the information theory, the smaller $I(L^1_{xy}|O_{xy})$ and $I(L^1_{xy}|P_{xy})$ are, the higher probability of a future link between nodes x and y tends to be. Therefore, we define the score as the negation of $I(L^1_{xy}|O_{xy})$ and $I(L^1_{xy}|P_{xy})$. For a simpler formalization, we define $\lambda = \lambda_2/\lambda_1$, and obtain

$$s^{NCI}_{xy} = -I(L^1_{xy}|O_{xy}) - \lambda I(L^1_{xy}|P_{xy}). \tag{23}$$

Data Description

Our experiments include 12 real-world networks drawn from disparate fields. Details are as follows and the basic structural features are presented in Table S1. The networks here are all regarded as undirected and unweighted networks. In order to better describe the perspectives of the networks, if a network is unconnected, we only consider the largest connected component.

- Karate [3] - A friendship network of a karate club at a US univercity in the 1970s.
- Everglades [4] - A network of foodweb in Everglades Graminoids during wet season.
- C.elegans (Celegans) [5] - A neural network of the nematode worm C.elegans.

Table S1. The basic structural features of twelve real-world networks. N and M are the numbers of nodes and links in the network, respectively. e is the network efficiency [12], defined as $e = \frac{2}{N(N-1)} \sum_{x,y \in V, x \neq y} d_{xy}^{-1}$, where d_{xy} is the shortest path distance between node x and node y. C and r are clustering coefficient [10] and assortative coefficient [13]. $\langle k \rangle$ and $\langle d \rangle$ denote the average degree and the average shortest distance. H is the degree heterogeneity, denoted as $H = \langle k^2 \rangle / \langle k \rangle^2$.

Networks	N	M	e	C	r	H	$\langle k \rangle$	$\langle d \rangle$
Karate	34	78	0.492	0.588	-0.476	1.693	4.588	2.337
Everglades	69	880	0.686	0.552	-0.298	1.275	25.507	1.612
Celegans	297	2148	0.445	0.308	-0.163	1.801	14.465	2.447
NS	379	941	0.203	0.798	-0.082	1.663	4.823	6.026
Email	1133	5451	0.300	0.254	0.078	1.942	9.622	3.603
PB	1222	16714	0.398	0.360	-0.221	2.971	27.355	2.735
Yeast	2375	11693	0.218	0.388	0.454	3.476	9.847	5.094
SciMet	2678	10368	0.257	0.203	-0.035	2.427	7.743	4.178
Kohonen	3704	12673	0.296	0.304	-0.121	9.317	6.843	3.669
EPA	4253	8897	0.236	0.136	-0.304	6.767	4.184	4.499
Power	4941	6594	0.063	0.107	0.004	1.450	2.669	18.985
Router	5022	6258	0.167	0.033	-0.138	5.503	2.492	6.448

- Network Science (NS) [6] - A network of coauthorships between scientists who are dong the research of network. The original network has 1589 nodes and 396 connected branches. Here we chose the giant component of this network which consists of 379 nodes and 914 links.
- Email [7] - A network of Alex Arenas's email.
- Political Blogs (PB) [8] - A network of the US political blogs.
- Yeast [9] - A protein-protein interaction network.
- Scientometrics (SciMet) [4] - A network of articles from or citing Scientometrics.
- Kohonen [4] - A network of articles with topic self-organizing maps or references to Kohonen.
- EPA [4] - A network of web pages linking to the website www.epa.gov.
- Power [10] - An electrical power grid of the western of US.
- Router [11] - The router-level topology of the Internet.

References

[1] Shannon CE (2001) A mathematical theory of communication. ACM SIGMOBILE Mobile Computing and Communications Review 5: 3–55.

[2] Cover TM, Thomas JA (2012) Elements of information theory. John Wiley & Sons.

[3] Zachary WW (1977) An information flow model for conflict and fission in small groups. Journal of Anthropological Research : 452–473.

[4] Pajek datasets. `http://vlado.fmf.uni-lj.si/pub/networks/data/`.

[5] White JG, Southgate E, Thomson JN, Brenner S (1986) The structure of the nervous system of the nematode caenorhabditis elegans. Philosophical Transactions of the Royal Society of London B, Biological Sciences 314: 1–340.

[6] Newman ME (2001) Clustering and preferential attachment in growing networks. Physical Review E 64: 025102.

[7] Duch J, Arenas A (2005) Community detection in complex networks using extremal optimization. Physical Review E 72: 027104.

[8] Adamic LA, Glance N (2005) The political blogosphere and the 2004 us election: divided they blog. In: Proceedings of the 3rd international workshop on Link discovery. ACM, pp. 36–43.

[9] Von Mering C, Krause R, Snel B, Cornell M, Oliver SG, et al. (2002) Comparative assessment of large-scale data sets of protein–protein interactions. Nature 417: 399–403.

[10] Watts DJ, Strogatz SH (1998) Collective dynamics of small-worldnetworks. Nature 393: 440–442.

[11] Spring N, Mahajan R, Wetherall D (2002) Measuring isp topologies with rocketfuel. In: ACM SIGCOMM Computer Communication Review. ACM, volume 32, pp. 133–145.

[12] Latora V, Marchiori M (2001) Efficient behavior of small-world networks. Physical Review Letters 87: 198701.

[13] Newman ME (2002) Assortative mixing in networks. Physical Review Letters 89: 208701.
